# A core outcome set for randomised controlled trials of physical activity interventions: development and challenges

**DOI:** 10.1186/s12889-022-12600-7

**Published:** 2022-02-24

**Authors:** Helen Crocker, Michele Peters, Charlie Foster, Nick Black, Ray Fitzpatrick

**Affiliations:** 1 Nuffield Department of Population Health, University of Oxford, Richard Doll Building, Old Road Campus, Headington, Oxford, OX3 7LF UK; 2 Quality Safety and Outcomes Policy Research Unit, Kent, UK; 3 Centre for Exercise, Nutrition and Health Sciences, School for Policy Studies, University of Bristol, Social Science Complex, 8 Priory Road, Bristol, BS8 1TZ UK; 4 Department of Health Services Research and Policy, London School of Hygiene and Tropical Medicine, 15-17 Tavistock Place, London, WC1H 9SH UK

**Keywords:** Core outcome set, Physical activity, Accelerometer, Device-based measurement, Measures

## Abstract

**Background:**

Core outcome sets are standardised sets of outcomes that should be collected and reported for all clinical trials. They have been widely developed and are increasingly influential in clinical research, but despite this, their use in public health has been limited to date. The aim of this study was to develop a core outcome set for public health trials evaluating interventions to promote physical activity in the general adult population.

**Methods:**

The core outcome set was developed using a three-stage approach: *stage one:* a review of literature to identify potential domains for inclusion in the core outcome set; *stage two:* a Delphi survey was carried out to reach consensus about which outcome domains to include in the core outcome set; and *stage three:* a second Delphi survey was conducted to determine how best to measure the outcome domains included in the core outcome set.

**Results:**

A classification of 13 outcome domains of physical activity was developed (*stage one*). Twenty people completed round one of the first Delphi survey (*stage two*), reaching a consensus to include two domains in the core outcome set, ‘device-based level of physical activity’ (80.0%, *n* = 16) and ‘health-related quality of life’ (70.0%, *n* = 14). No further consensus on the remaining outcome domains was reached in round two. Nineteen people completed the second Delphi survey (*stage three*). Participants rated the accelerometer (mean rating = 3.89, on a scale of 1 (do not recommend) to 5 (highly recommend)) as the best device to measure level of physical activity, and the EQ-5D (73.7%, *n* = 14) as the most appropriate measure of health-related quality of life.

**Conclusions:**

This study has made progress towards the development of a core outcome set for use in physical activity trials, however, there was limited consensus about which domains to include. The development of the core outcome set was challenged by the need for trial-specific outcomes, and the complexities of collecting, processing and reporting device-based data.

**Supplementary Information:**

The online version contains supplementary material available at 10.1186/s12889-022-12600-7.

## Introduction

Physical activity is an important determinant of health, but large proportions of populations remain physically inactive. In producing research evidence through trials of interventions to reduce inactivity, one problem is a lack of consensus about which measures of physical activity to use [1–3]. This study examines whether the methodology of core outcome sets can solve this problem.

Core outcome sets are agreed, standardised sets of outcomes that represent the minimum group of outcomes that should be collected and reported for all clinical trials and related evaluative research for a specific health condition [4]. They have been widely developed and are increasingly influential in clinical research. Their use is intended to produce authoritative agreement on a set of outcome measures to be used in all trials, thus reducing heterogeneity of outcomes measurement [5]. The result of such standardisation is to make systematic reviews and meta-analyses of trials easier to facilitate the interpretation of available evidence to inform public policy. The method of core outcome sets is designed to reach agreement about two different levels in outcome measurement: (i) broad domains or areas that need to be assessed in trials, and (ii) specific measures that can be recommended for each identified domain or area.

Initiatives such as Core Outcome Measures in Effectiveness Trials (COMET) aim to standardise measurement across clinical trials by supporting the development and use of core outcome sets and, as part of their remit, they maintain a database of core outcome sets [6]. Core outcome sets were originally developed for use in clinical trials for specific health conditions (e.g. asthma and Parkinson’s disease), but are now applied to research beyond clinical trials, as well as in clinical practice, with standard sets developed by ICHOM (International Consortium for Health Outcomes Measurement) of particular note [7]. Furthermore, more general sets are starting to emerge, for example, core outcome sets addressing older person health and overall adult health have recently been developed (see [8]).

Despite the proliferation of core outcome sets, to date, few have been developed for public health research, including physical activity. There is great heterogeneity in trials to evaluate interventions to promote physical activity, in terms of both the variety of constructs as well as by what technology [1, 9, 10]. This shows that there is fundamental non-comparability of constructs measured between trials, rendering systematic reviews and meta-analyses of comparative evidence problematic. A clear and consistent trend towards increasing range and methods of device-based measures [1], will only exacerbate this issue. As such, many reviews conclude that there is a need for consensus around a consistent approach to collecting and reporting data to allow comparisons across instruments [2]. The NIHR also recently called for greater consistency in approaches to the measurement of physical activity to better inform decision makers and the implementation of research [11]. Despite increasing pleas to standardise the measurement of physical activity in light of this proliferation of methods and measures, no standardisation has emerged. Few core outcome sets have been proposed or developed in the field of physical activity, and those that have, have focused on disease-specific populations (e.g. dementia [12], groups of diseases (e.g. musculoskeletal diseases [13]) or specific settings (e.g. primary schools [14])).

For the above reasons, the aim of this study was to assess the feasibility of developing a core outcome set for public health trials to promote physical activity in the general adult population, using the standard methods of core outcome sets. This was to be achieved by the following four objectives: (1) To identify all potential outcome domains for the core outcome set following a review of relevant literature; (2) To reach consensus on which outcome domains should be included in the core outcome set through a consensus development process; (3) To identify potential measurement tools for each of the outcome domains for which consensus was reached in objective 2; and (4) To achieve consensus on the selection of measurement tools for each outcome domain within the core outcome set, through a consensus development process.

## Methods

**Fig. 1:**
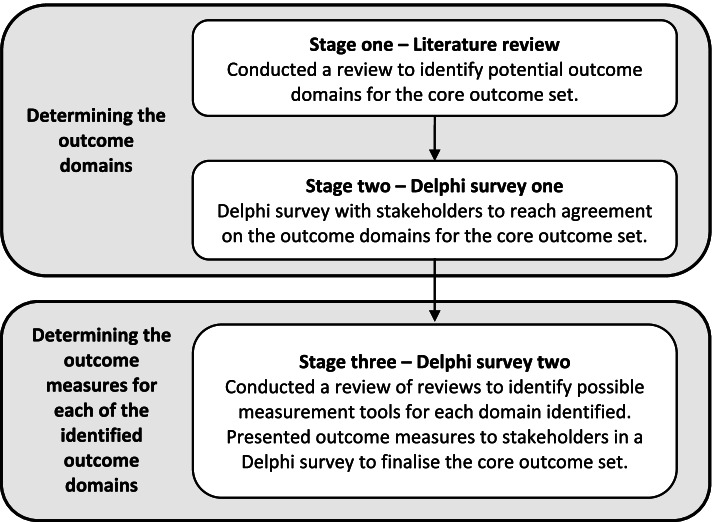
Stages in the development of the core outcome set

The development of the core outcome set was carried out in the following three stages (see Fig. 1), which broadly follows the methodology set out by the COMET initiative [15]: *stage one* – a review of the literature to identify potential domains for inclusion in the core outcome set; *stage two* – a consensus development process to determine the outcome domains to include in the core outcome set; and *stage three* – a literature review followed by a further consensus development process to determine how the outcome domains identified in *stage two* should be measured. Each of these stages will now be explained in greater detail.

### Stage one – literature review to identify outcome domains of physical activity

A classification of physical activity domains was developed following a review of trials of physical activity interventions. The aim was to identify the main outcome domains collected and reported in trials assessing physical activity interventions. Firstly, relevant reviews of trials of physical activity interventions were identified using the following search criteria to search PubMed and Google Scholar: “physical activity” OR “physical fitness” OR “health promotion” AND review* OR evaluation OR “meta-analysis” AND ((randomised OR randomized) AND trial) OR RCT OR “clinical trial*” NOT children OR paediatric OR pediatric. Only interventions for the general adult population were included. Those focused on children or people with specific conditions, or protocols, were excluded. Included papers were reviewed to identify outcome domains measured, following which, researchers (HC, RF, MP) developed a classification of physical activity outcome domains. The classification was refined following further work reviewing outcomes of primary studies of randomised controlled trials to assess interventions for physical activity improvement (literature searches were conducted as above less the references to “review*”, “evaluation” and “meta-analysis”). The same inclusion and exclusion criteria as above were applied. All authors agreed the final classification.

### Stage two – determining the outcome domains

The purpose of stage two was to determine which outcome domains should be included in the core outcome set. This was achieved through an electronic Delphi survey, comprised of two rounds. In round one (July – August 2020), participants were presented with a list of 13 outcome domains commonly measured in clinical trials of physical activity (identified in *stage one*), together with a definition of each domain. Participants were asked to rate the importance of each outcome domain on a scale of 1 to 9, where 1–3 signified ‘limited importance’, 4–6 was ‘important but not critical’ and 7–9 was ‘critical importance’. The outcome domains were presented in alphabetical order. Participants were also invited to give reasons for their ratings and suggest any additional outcome domains for inclusion in the next round. Agreement to include a domain in the core outcome set followed criteria set out by COMET [15, 16], with agreement considered to be reached if ≥70% of participants rated the domain between 7 and 9 and < 15% rated the domain between 1 and 3. Agreement to exclude a domain from the core outcome set was considered to be reached if ≥70% rated the domain between 1 and 3 and < 15% rated the domain between 7 and 9. For domains not meeting inclusion or exclusion criteria, no consensus was reached.

Following completion of the first round, a second round was held (October – November 2020), following the same methodology for the domains for which no consensus was reached. Participants were presented with a list of outcome domains and their definitions, together with a summary of results from round one (the average (mean) rating for each domain, the distribution of ratings (i.e. the percentage of participants rating the domain 1–3, 4–6, and 7–9), and the rating the participant gave in the previous round). Participants were asked to re-rate the outcome domains in light of the round one results and comment on their reasons for rating the outcome domains as they did. The same criteria as round one was used to judge whether a consensus for each outcome domain was reached.

### Stage three – determining the measurement instruments for the agreed outcome domains

**Fig. 2:**
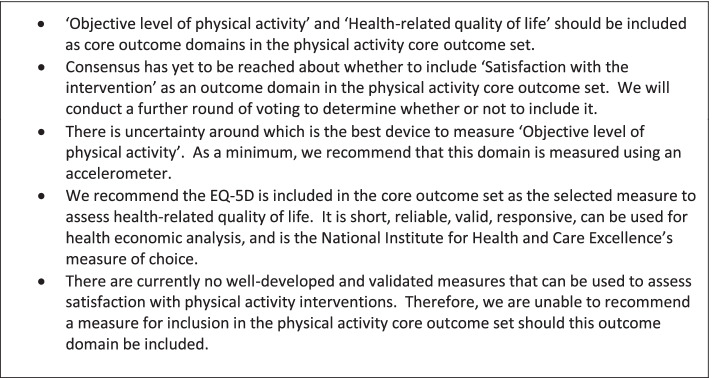
Recommendations for the physical activity core outcome set

The purpose of stage three was to determine which outcome measures should be included in the core outcome set for each of the agreed outcome domains identified in *stage two*. Scoping reviews were conducted for each outcome domain that reached or were close to consensus to identify and evaluate available measures for the assessment of each domain. The reviews informed recommendations about how best to measure each domain, and were agreed by all authors (see Fig. 2 for recommendations).

For *Stage three* (March – April 2021), participants were given a report detailing the study background, the results of *stage two*, brief summaries of the literature on available measures and key measurement issues for the domains of interest, and the authors’ recommendations for the core outcome set (see Additional file 1). Participants were asked to read the report before completing a second electronic Delphi survey, consisting of up to six questions. The first question asked participants to rate a list of device-based measures of physical activity from 1 (do not recommend) to 5 (highly recommend), and the mean for each option was calculated. The remaining five questions asked participants whether they were in agreement with five separate statements about the measurement of the included outcome domains and a further proposed outcome domain (see Table 1 for statements).

**Table 1:** Stage three Delphi survey statements

*Do you agree or disagree with the following statement:*	
*Device-based level of physical activity*	
Raw accelerometer data should be collected and made publicly accessible to maximise the potential for future data analysis and thus, the ability to compare data across studies.	
Physical activity-related energy expenditure is the key construct to measure using devices that assess physical activity in future public health trials.	
*Health-related quality of life*	
The EQ-5D is the most appropriate measure for health-related quality of life for inclusion in a core outcome set for physical activity trials.	
*Satisfaction with the intervention*	
‘Satisfaction with the intervention’ is an essential domain for a core outcome set for physical activity trials.	
(Only asked if the participant agreed with the inclusion of the outcome domain) There is no suitable measure to assess ‘satisfaction with the intervention’ in physical activity trials.	

### Recruitment

The following groups were invited to participate in the study: academics with an interest in physical activity or outcomes measurement; professionals working in the field of public health; health care professionals; and lay people aged 18 or over. Professionals were identified through an Internet search and snowballing. Lay people were recruited through the Quality, Safety and Outcomes Policy Research Unit’s Public Involvement Research Advisor Network, and snowballing. All participants were provided with a participant information sheet prior to taking part and gave informed electronic consent to participate in the study.

This study was reviewed by, and received ethics clearance through, the University of Oxford Central University Research Ethics Committee (Reference number: R70120/RE001).

### Patient and public involvement

A Patient and Public Involvement (PPI) advisor (MB) was a member of the advisory group who met in the preliminary stages of the study to discuss the feasibility of the core outcome set approach in public health. Two PPI advisors (AA and MB) were involved in discussions to agree an approach to the study and the classification of physical activity outcome domains. Furthermore, as indicated above, four lay people took part in the Delphi surveys to help ensure that the resulting core outcome set reflects what matters most to adults in the general population, i.e. those targeted by the physical activity behavioural interventions relevant to this study.

## Results

The results of the literature review (stage one) and Delphi surveys (stages two and three) are presented below, together with the final core outcome set.

### Stage one – literature review to identify outcome domains of physical activity

A literature review to identify potential outcome domains of physical activity, followed by discussion between authors, led to the final classification of the following 13 outcome domains: adverse events; biophysical health; cost-effectiveness; health-related quality of life; device-based level of physical activity; self-reported level of physical activity; motivation; other health behaviours; physical fitness; physical function; satisfaction with the intervention; sedentary behaviour; and self-efficacy. Of particular note, and given the debates around the measurement of the level of physical activity, it was decided to create two outcome domains for ‘level of physical activity’, with one focused on data collected through self-report measures and the other on device-based measures. Each domain was defined by the authors to support the consensus development process (see Table 2 for definitions).

**Table 2:** Classification of physical activity outcome domains

Outcome domain	Definition
Adverse events	An untoward health or medical occurrence in an individual (e.g. injuries, pain, falls). The adverse event does not necessarily have a causal relationship with the trial intervention.
Biophysical health	Health as defined through biological or physical measures; properties; and/or norms. Examples include signs (e.g. blood pressure), symptoms (e.g. pain) and comorbidities.
Cost-effectiveness	Cost-effectiveness is the degree to which something is effective or productive in relation to its cost i.e. good value for money.
Health-related quality of life	A broad multidimensional concept that focuses on an individual’s self-perceived and subjective health, and impact of health and disease (including symptoms) on day to day life. Dimensions include physical, mental, emotional and social functioning; and impacts of these dimensions associated with an individual’s perceptions such as health risks and conditions, functional status, and social support.
Level of physical activity (device-based)	The objective (i.e. measured by external methods such as a pedometer) amount of physical activity or bodily movement that engages skeletal muscles and that leads to energy expenditure.
Level of physical activity (self-report)	The subjective or self-reported amount of physical activity or bodily movement that engages skeletal muscles and that leads to energy expenditure.
Motivation	Reasons for individual’s to act or behave in a particular way to achieve goals; fulfil basic physical (e.g. hunger) and psychological needs (e.g. social contact); or uphold values (i.e. things an individual considers important such as family and health). It can be intrinsic, which means doing an activity for its inherent satisfactions (e.g. feeling of enjoyment). It can also be extrinsic, which means doing an activity for instrumental reasons, or to obtain some outcome separable from the activity per se *(*e.g. *gaining a tangible reward).*
Other health behaviours	Health behaviours other than physical activity that may vary or change as a result of engaging in physical activity. These could be lifestyle behaviours (e.g. diet, smoking); illness related behaviours (e.g. health care and/or medication use); or health outcomes (e.g. sleep quality).
Physical fitness	The condition of an individual being physically strong and healthy or in other words, achieving positive health (i.e. level of health and well-being beyond the absence of illness). It can be health-related (cardio-respiratory endurance; muscular endurance and strength; body composition; flexibility; and strength) or skills-related (e.g. agility; balance; coordination; speed; power; and reaction time).
Physical function	The ability to perform basic and instrumental activities of daily living including tasks such as dressing and bathing, or activities such as walking a short distance to exercising vigorously.
Satisfaction with intervention	The extent to which study participants experience and perceive the physical activity intervention as positive.
Sedentary behaviour	Any waking behaviour characterized by low energy expenditure, while in a sitting, reclining or lying posture. Common examples include TV viewing, desk-based occupations, computer use, passive commuting (car, taxi), reading, and playing board games.
Self-efficacy	An individual’s belief about their capabilities to respond to events and to exercise control over their own activities in ways that influence events that effect their life.

### Stage two – determining the outcome domains

Forty-one people (36 professionals and five lay people) were invited to take part in a Delphi survey to give their opinions about which outcome domains should be included in the core outcome set. Twenty people (16 professionals and four lay people) consented to participate and completed the first round. All participants were from high-income countries. The majority of professional participants were academic researchers with an interest in physical activity, outcomes measurement, or both. Two academics were current or past health care professionals, and one participant was a health care professional only. In terms of career stage, academics were at various stages, but there was a skew towards those holding higher positions. Of the four lay participants, three were female and one male, and their ages ranged from ‘18–29’ to ‘60–69’.

A summary of participants’ ratings of the importance of outcome domains can be found in Table 3. At least 70% of participants rated ‘Level of physical activity (device-based)’ and ‘Health-related quality of life’ as domains of critical importance, meeting the criteria for inclusion in the core outcome set. No consensus was reached for the remaining 11 outcome domains and therefore a second round was conducted. No new outcome domains were added for consideration in the second round.

**Table 3:** Results of the stage two Delphi survey, rounds 1 and 2

Domain		Rated 1–3 (limited importance)	Rated 4–6 (important but not critical)	Rated 7–9 (critical importance)
	Round	n (%)	n (%)	n (%)
Level of physical activity (device-based)	1	1 (5.0)	3 (15.0)	**16 (80.0)**
	2	–	–	–
Health-related quality of life	1	0 (0.0)	6 (30.0)	**14 (70.0)**
	2	–	–	–
Other health behaviours	1	1 (5.0)	5 (26.0)	13 (68.0)
	2	4 (19.1)	7 (33.3)	10 (47.6)
Satisfaction with intervention	1	1 (5.0)	6 (32.0)	12 (63.0)
	2	1 (4.8)	6 (28.6)	14 (66.7)
Biophysical health	1	1 (5.0)	7 (35.0)	12 (60.0)
	2	2 (9.5)	9 (42.9)	10 (47.6)
Level of physical activity (self-report)	1	2 (10.0)	6 (30.0)	12 (60.0)
	2	4 (19.1)	7 (33.3)	10 (47.6)
Cost-effectiveness	1	1 (5.0)	8 (40.0)	11 (55.0)
	2	1 (4.8)	10 (47.6)	10 (47.6)
Adverse events	1	4 (20.0)	5 (25.0)	11 (55.0)
	2	1 (4.8)	9 (42.9)	11 (52.4)
Self-efficacy	1	2 (11.0)	7 (37.0)	10 (53.0)
	2	5 (23.8)	7 (33.3)	9 (42.9)
Sedentary behaviour	1	3 (16.0)	7 (37.0)	9 (47.0)
	2	4 (19.1)	6 (28.6)	11 (52.4)
Motivation	1	1 (5.0)	10 (53.0)	8 (42.0)
	2	3 (14.3)	11 (52.4)	7 (33.3)
Physical fitness	1	2 (11.0)	9 (47.0)	8 (42.0)
	2	2 (9.5)	11 (52.4)	8 (38.1)
Physical function	1	0 (0.0)	12 (63.0)	7 (37.0)
	2	0 (0.0)	10 (47.6)	11 (52.4)

Note: Round 1, *n* = 20. One individual did not rate all of the outcome domains in the first round due to technical issues. Round 2, *n* = 21

Thirty-six people (32 professionals and four lay people) were invited to take part in a second round (seven participants had opted out of participating in the research and were not invited to take part in the second round, and two newly identified participants were invited (both academic researchers with an interest in physical activity)). The remaining 11 outcome domains were presented to participants to rate their importance. Twenty-one people took part (17 professionals and four lay people), 17 of which had participated in the first round. No consensus was reached for any of the 11 outcome domains (see Table 3), however, ‘satisfaction with the intervention’ was close to reaching consensus, with 66.7% of participants rating it as critically important.

### Stage three - determining the measurement instruments for the agreed outcome domains

Thirty-six people were invited to take part in a second Delphi survey (the same participants were invited to participate as were invited in to the second round of the first Delphi survey). Of the 36 participants invited to participate, 19 (15 professionals and four lay people) completed the second Delphi survey to provide their opinions on the best measurement tools and approaches for the identified domains. The distribution of professional roles was the same as *stage two*. Overall, 15 participants (11 professionals and four lay people) took part in all stages of both Delphi surveys. As ‘satisfaction with the intervention’ was close to consensus in *stage two*, further opinions about the inclusion of this domain within the core outcome set were sought as part of this survey. The results for each outcome domain are presented in turn.

### Device-based level of physical activity

Overall, respondents rated the accelerometer (mean rating = 3.89, score range 1–5) as the best device to include in the core outcome set for measuring level of physical activity, followed by a multi-sensor device to include an accelerometer (mean rating = 3.32), an accelerometer plus heart rate monitor (mean rating = 3.11), and a multi-sensor device without an accelerometer (mean rating = 2.11). Nine respondents provided reasoning for their ratings. Several respondents highlighted the importance of the acceptability of devices to trial participants, with compliance likely to be increased through the use of accelerometers only, particularly those that tolerate water immersion. Multiple sensors and heart rate monitors were considered less acceptable to participants, particularly when worn for longer times. Furthermore, one participant considered the value added by a multi-sensor device to be limited, while another cautioned that their use on a larger scale could be too expensive.

There was support (68.4%, *n* = 13) for raw accelerometer data to be collected and made publicly accessible, however, this did not reach the minimum level of agreement for consensus. There was limited agreement (42.1%, *n* = 8) that Physical Activity-related Energy Expenditure (PAEE) should be considered the key construct to be measured by devices to assess level of physical activity.

### Health-related quality of life

There was consensus (73.7%, *n* = 14) that the EQ-5D should be included in the core outcome set to assess health-related quality of life, with only five respondents (26.3%) disagreeing and suggesting alternative measures, such as the SF-36 [17], SF-12 [18], or the Warwick-Edinburgh Mental Wellbeing Scale (WEMWBS) [19].

### Satisfaction with the intervention

While many (68.4%, *n* = 13) considered ‘satisfaction with the intervention’ an essential domain for inclusion in the core outcome set, the minimum level of agreement for consensus was not reached. Of those who wished to include the domain, there was agreement (84.6%, *n* = 11) that there is currently no suitable measure available to assess the domain.

The final core outcome set is shown in Table 4.

**Table 4:** The final core outcome set for physical activity trials

Outcome domain	Measurement tool
Device-based level of physical activity	Accelerometer
Health-related quality of life	EQ-5D

## Discussion

In this study assessing the feasibility of applying core outcome set methodology to the field of physical activity, participants reached limited agreement about which outcome domains should be included in a core outcome set for physical activity trials. Out of 13 relevant domains, professionals and lay people agreed the inclusion of just two, ‘device-based level of physical activity’ and ‘health-related quality of life’, with no consensus reached for the remaining domains. This suggests that this approach had some success within this field as there was strong support for the inclusion of two outcome domains. However, it is worth noting that the resulting core outcome set was less comprehensive than is typical (as an example, hip fracture and neonatal research core outcome sets included five and 12 outcome domains respectively [20, 21]). This discrepancy is likely due to a tendency for core outcome sets to focus on specific populations and/or contexts, whereas the core outcome set described in this research is intended for a broader population. While the core outcome set has been developed for use in randomised controlled trials, it would also be applicable to well-designed observational studies.

A common theme raised by participants throughout the study was the need for chosen outcome domains to reflect the study’s research question, aims, and target population. As such, many participants found the task of rating outcome domains, as part of the *stage two* Delphi survey, a difficult task in the absence of the context of a specific trial, with concerns that the resulting core outcome set may be “overly blunt”. This theme is reflected in the literature, where there is a general consensus that measures selected should be specific to the behaviour of interest and the type of data intended to be collected [2, 22]. Conversely, as a result of the large variety of outcomes reported across trials [1, 9, 10], there are pleas for standardisation in order to produce evidence for better practice [23]. Hence, there appears to be a fundamental tension between measuring outcomes relevant to individual trials and enabling comparisons of results across trials. This may go some way towards explaining why the resulting core outcome set was limited to two outcome domains.

Participants reached agreement that the accelerometer is the best device for the measurement of level of physical activity to include in the core outcome set. However, challenges around how best to collect, process and report such data remain [23]. A systematic review of measurement and data collection and processing practices associated with the use of accelerometers found major variations in terms of device placement, sampling frequency, wear-time, what constitutes a valid day and a valid week, cut-points for sedentary time and physical activity intensity classification, and algorithms to estimate physical activity-related energy expenditure [24]. Such variations have been found to have an impact on the interpretation of the data collected [24], for example, the treatment of interruptions (i.e. breaks in exercise) or the classification of data into different physical activity groups by epoch length (i.e. the interval at which accelerometer data is recorded) has been found to affect the estimation of physical activity [25, 26]. Furthermore, analytical approaches to the analysis of accelerometer data are progressing rapidly. As a step towards addressing these issues, Migueles et al. [24] recommend the collection of raw accelerometer data in order to maximise the potential for any future data analysis. This suggestion was put to participants as part of this study, but there was no agreement that raw accelerometer data should be collected. Furthermore, the comparability of data between studies is further complicated by poor reporting of data collection and processing practices [24, 27].

While there was consensus among participants for the inclusion of device-based measures of level of physical activity in the core outcome set, it is of note that no such agreement for self-reported levels of physical activity was reached. This is in contrast to those such as Skender et al. [28] and Falck et al. [3] who advise that studies collect and report both device-based and self-report measures of physical activity to obtain the most complete information about physical activity. However, this result may be reflective of greater variability found in measurement properties of self-report measures than of device-based measures [2, 3].

Moving forward, further work is needed to explore the viability of the standardisation of data collection, processing and reporting practices of accelerometer data. This is an important goal if comparative evidence of performance to inform recommendations is to be achieved. While there was limited agreement regarding which outcome domains to include in the core outcome set, this offers researchers the flexibility to select trial-specific outcomes. A full core outcome set may not be applicable in this field, and it may be that a more flexible core outcome set offering guidance for different types of trial or trial aims may be more relevant. This would allow for the inclusion of trial-specific outcomes while also encouraging standardisation across trials of a similar nature.

There are some limitations to this study. The recruitment of professionals and lay people to take part in the Delphi surveys took place between July 2020 and April 2021, during the Covid-19 pandemic. Due to the pressures on health care and public health professionals as a result of the pandemic, it was felt inappropriate to recruit many members of these groups in to the study and therefore, panel members from these groups were limited. It is possible that members of these groups hold different views than others in the study, and if so, such views would be underrepresented. Secondly, it is possible that searching additional databases as part of the initial review to identify outcome domains may have identified further literature and outcome domains of interest. However, the likelihood of missing important outcome domains was minimised as participants of the Delphi surveys were encouraged to suggest outcome domains that they felt were important, but that may have been missed from the classification. Finally, it is possible that further consensus may have been reached through the more constructive dynamic of an in-person consensus meeting.

## Conclusions

There is a fundamental tension between the desire to attain trial-specific outcomes and the standardisation of outcomes across trials to more easily synthesise evidence to inform policy. This is further complicated by the complexities of collecting, processing, and reporting device-based data. These issues make the application of core outcome set methodology to the field of physical activity challenging. While this study has progressed the development of a core outcome set for physical activity trials, further work is required to standardise accelerometer data. It may be that the core outcome set needs to have different sets of guidance dependent on the trial type. This would be a compromise on the traditional core outcome set, but would be a step towards standardisation and the production of better evidence.

## Supplementary Information

**Additional file 1.** Stage three report for Delphi panel members

## Data Availability

The dataset supporting the conclusions of this article is included within the article and its additional file.

## Abbreviations

COMET: Core Outcome Measures in Effectiveness Trials
COS: Core Outcome Set
ICHOM: International Consortium for Health Outcomes Measurement

## References

[1] Silfee VJ, Haughton CF, Jake-Schoffman DE, Lopez-Cepero A, May CN, Sreedhara M (2018). Objective measurement of physical activity outcomes in lifestyle interventions among adults: a systematic review. Prev Med Rep.

[2] Dowd KP, Szeklicki R, Minetto MA, Murphy MH, Polito A, Ghigo E (2018). A systematic literature review of reviews on techniques for physical activity measurement in adults: a DEDIPAC study. Int J Behav Nutr Phys Act.

[3] Falck RS, McDonald SM, Beets MW, Brazendale K, Liu-Ambrose T (2016). Measurement of physical activity in older adult interventions: a systematic review. Br J Sports Med.

[4] COMET Initiative: About COMET. http://www.comet-initiative.org/about/overview. Accessed 21 July 2021.

[5] Webbe J, Sinha I, Gale C (2018). Core outcome sets. Arch Dis Child Educ Pract Ed.

[6] COMET Initiative: Core outcome measures in effectiveness trials. https://www.comet-initiative.org/. Accessed 21 July 2021.

[7] ICHOM: International Consortium for Health Outcomes Measurement. https://www.ichom.org/. Accessed 21 July 2021.

[8] ICHOM: Standard sets. https://www.ichom.org/standard-sets/. Accessed 23 July 2021.

[9] Foster C, Richards J, Thorogood M, Hillsdon M. Remote and web 2.0 interventions for promoting physical activity. Cochrane Database Syst Rev. 2013. 10.1002/14651858.CD010395.pub2. 10.1002/14651858.CD010395.pub2 PMC9674455 24085594

[10] Stockwell S, Schofield P, Fisher A, Firth J, Jackson SE, Stubbs B (2019). Digital behavior change interventions to promote physical activity and/or reduce sedentary behavior in older adults: a systematic review and meta-analysis. Exp Gerontol.

[11] National Institute for Health Research (NIHR). Moving matters: interventions to increase physical activity. NIHR Dissemination Centre. 2019. 10.3310/themedreview-03898.

[12] Gonҫalves A, Samuel D, Ramsay M, Demain S, Marques A (2020). A core outcome set to evaluate physical activity interventions for people living with dementia. Gerontologist..

[13] Thompson A, Mallett R, Harrop D, Asif PT, McLean S. Development of a core set of outcomes for exercise and physical activity schemes. Physiotherapy. 2019. 10.1016/j.physio.2018.11.264.

[14] Foley KA, Venkatraman T, Ram B, Ells L, Van Sluijs E, Hargreaves DS, et al. Protocol for developing a core outcome set for evaluating school-based physical activity interventions in primary schools. BMJ Open. 2019. 10.1136/bmjopen-2019-031868. 10.1136/bmjopen-2019-031868 PMC6937029 31852702

[15] Williamson PR, Altman DG, Bagley H, Barnes KL, Blazeby JM, Brookes ST, et al. The COMET handbook: version 1.0. Trials. 2017. 10.1186/s13063-017-1978-4. 10.1186/s13063-017-1978-4 PMC5499094 28681707

[16] Williamson PR, Altman DG, Blazeby JM, Clarke M, Devane D, Gargon E, et al. Developing core outcome sets for clinical trials: issues to consider. Trials. 2012. 10.1186/1745-6215-13-132. 10.1186/1745-6215-13-132 PMC3472231 22867278

[17] Ware JE, Sherbourne CD (1992). The MOS 36-item short-form health survey (SF-36). I. Conceptual framework and item selection. Med Care.

[18] Ware JE (1995). SF-12: how to score the SF-12 physical and mental health summary scales.

[19] Tennant R, Hiller L, Fishwick R, Platt S, Joseph S, Weich S, et al. The Warwick-Edinburgh mental well-being scale (WEMWBS): development and UK validation. Health Qual Life Outcomes. 2007. 10.1186/1477-7525-5-63. 10.1186/1477-7525-5-63 PMC2222612 18042300

[20] Haywood KL, Griffin XL, Achten J, Costa ML. Developing a core outcome set for hip fracture trials. Bone Joint J. 2014. 10.1302/0301-620X.96B8.33766. 10.1302/0301-620X.96B8.33766 25086115

[21] Webbe JWH, Duffy JMN, Afonso E, Al-Muzaffar I, Brunton G, Greenough A, et al. Core outcomes in neonatology: development of a core outcome set for neonatal research. Arch Dis Child Fetal Neonatal Ed. 2019. 10.1136/archdischild-2019-317501. 10.1136/archdischild-2019-317501 PMC7363790 31732683

[22] Sylvia LG, Bernstein EE, Hubbard JL, Keating L, Anderson EJ (2014). A practical guide to measuring physical activity. J Acad Nutr Diet.

[23] Jake-Schoffman D, Silfee V, Sreedhara M. Reporting of physical activity device measurement and analysis protocols in lifestyle interventions. Am J Lifestyle Med. 2019. 10.1177/1559827619862179. 10.1177/1559827619862179 PMC8669894 34916889

[24] Migueles JH, Cadenas-Sanchez C, Ekelund U, Delisle Nystrӧm C, Mora-Gonzalez J, Lӧf M (2017). Accelerometer data collection and processing criteria to assess physical activity and other outcomes: a systematic review and practical considerations. Sports Med.

[25] Ayabe M, Kumahara H, Morimura K, Tanaka H (2013). Epoch length and the physical activity bout analysis: an accelerometry research issue. BMC Res Notes..

[26] Ayabe M, Kumahara H, Morimura K, Tanaka H (2014). Interruption in physical activity bout analysis: an accelerometry research issue. BMC Res Notes.

[27] Montoye AHK, Moore RW, Bowles HR, Korycinski R, Pfeiffer KA (2018). Reporting accelerometer methods in physical activity intervention studies: a systematic review and recommendations for authors. Br J Sports Med.

[28] Skender S, Ose J, Chang-Claude J, Paskow M, Brühmann B, Siegel EM, et al. Accelerometry and physical activity questionnaires – a systematic review. BMC Public Health. 2016. 10.1186/s12889-016-3172-0. 10.1186/s12889-016-3172-0 PMC4910242 27306667

