# Supplementary material for: A core outcome set for randomised controlled trials of physical activity interventions: development and challenges
Source: BMC Public Health. 2022 Feb 24;22:389. doi: 10.1186/s12889-022-12600-7 (PMC8866915; doi:10.1186/s12889-022-12600-7)
Supplement: Supplementary file 1 — Additional file 1. Stage three report for Delphi panel members [file 12889_2022_12600_MOESM1_ESM.pdf]

The development of a core outcome set for randomised controlled trials of individual-level physical activity interventions

Reaching consensus about the measurement of physical activity domains

Plain English Summary

A key goal of public health is to increase physical activity among adults. To achieve this, various different approaches are developed (e.g. digital apps, support groups) and their effectiveness assessed in randomised controlled trials, where outcomes between different groups are compared. Examples of outcomes that might be measured include: level of physical activity, cardio-respiratory fitness, and wellbeing. However, it is often difficult for researchers to agree on which outcomes to measure. The purpose of this study is to develop consensus on which outcomes should be measured in randomised controlled trials of physical activity interventions, and determine how these outcomes should be measured (i.e. which measurement tools). This will result in the development of a 'core outcome set'. A core outcome set is a list of outcomes that experts have agreed should be collected and reported, as a minimum, for a specific health condition or area of health and for a specific purpose, e.g. clinical trials or clinical practice. They define *what* should be measured (which outcome domains) and *how* they should be measured (which measurement tools). The development and use of core outcome sets is intended to help standardise outcomes measured in research, thus allowing the results of studies to be compared and combined more easily.

In the first stage of this study we carried out a survey asking experts and members of the public to rate the importance of 13 outcome domains commonly measured in clinical trials of physical activity. A consensus was reached that 'Objective level of physical activity' and 'Health-related quality of life' should be included in the core outcome set. There was uncertainty about whether a third domain 'Satisfaction with the intervention' should also be included, and the other domains were excluded.

In this stage, we will conduct a second survey to reach consensus about how best to measure each outcome domain included in the core outcome set, while also seeking further views about whether or not to include the 'Satisfaction with the intervention' domain in the final core outcome set. To inform the second survey, we have reviewed the literature around the measurement of the three identified domains to reach recommendations about how best to measure each. Next, the key issues identified within the literature will be summarised for each of the identified domains in turn.

Domain 1 – Objective level of physical activity

The objective measurement of physical activity is the measurement of bodily movements using external methods or devices. Different aspects of these movements can be measured,

namely their intensity, duration, frequency, and type of activity. Physical activity is increasingly measured using one or more of the following devices: pedometers, accelerometers, heart rate monitors, and multi-sensor systems (i.e. a system comprised of two or more devices). Further information about each of these devices can be found on pages 7-8).

Studies have found large variations in how devices are used to collect and process data, for example, where the device is placed on the body, for how long the device is worn, and the processes applied to estimate the amount of energy expended on physical activity. Furthermore, it has been shown that differences in how the data is processed can affect how it is interpreted, for example, the way breaks in exercise are handled affects the estimation of time spent doing moderate to vigorous physical activity. As there is no standard for how data should be collected, processed or reported, and as approaches to analysing data rapidly progress, it is recommended that raw data is collected to allow for the data to be analysed in different ways in the future. In addition, making the raw data publicly accessible will help researchers to compare data across studies.

In the literature there is a general consensus that the selection of a measurement tool for assessing physical activity should be based on the type of activity being studied, while also considering other factors, such as the feasibility, reliability and cost of different devices. However, several studies have found multi-sensor devices or accelerometers together with another device, such as a heart rate monitor, perform well.

As well as a large variety of devices, clinical trials also report a large variety of outcomes. This makes comparing outcomes between trials problematic, for example, number of steps cannot easily be compared with number of minutes spent doing moderate intensity activity. Some outcomes reported are specific to the activity, for example, minutes of walking per day, and are therefore not suitable for inclusion in the core outcome set. More general outcomes, such as physical activity-related energy expenditure (PAEE), apply to all types of activity and therefore are appropriate for inclusion in the core outcome set. PAEE estimates the amount of energy used to carry out physical activities such as walking and climbing stairs. Data collected through devices such as accelerometers, can be used to estimate PAEE, however, challenges remain about how best to do this.

Domain 2 – Health-related quality of life

Health-related quality of life is typically measured by patient-reported outcome measures (PROMS), short questionnaires which ask people about their physical and mental well-being, symptoms, social functioning and so on. Outcome measures included in the core outcome set should be relevant for all adults and therefore, the quality of life measure chosen should be relevant for both those that are healthy and those with health problems. Two commonly used measures meeting this criteria are the SF-36 and the EQ-5D.

The SF-36 asks 36 questions about the following: bodily pain; general health perceptions; mental health; physical functioning; role limitations due to emotional health problems; role limitations due to physical health problems; social functioning; and vitality. The answers given can be scored to create a range of summary scores. The SF-36 has been widely used and found to be a valid and reliable questionnaire. A shorter version of the questionnaire, the SF-12, was derived from the SF-36, and has 12 questions. However, in trials of physical activity, it appears to be used far less frequently than the SF-36.

The EQ-5D has five questions asking about: anxiety/depression; pain; self-care; mobility; and usual activities. In addition to the five questions, people are asked to rate their health on a scale from 0 to 100. It is possible to convert these answers (i.e. people's health state) in to a single number which aids decisions about health care to be made, including health economic analyses. It has been widely used and found to be a valid and reliable questionnaire. It is also the measure of choice for the National Institute for Health and Care Excellence (NICE), a body sponsored by the Department of Health and Social Care which aims to improve health outcomes.

A search of the literature found that the SF-36 and its derivatives, and the EQ-5D appear to be the most used health-related quality of life questionnaires in physical activity clinical trials. A review paper looking at studies exploring quality of life in relation to physical activity found that the SF-36 and its derivatives were the most commonly used questionnaires to assess health-related quality of life.

Domain 3 – Satisfaction with the intervention

The domain 'Satisfaction with the intervention' captures participants' experience of the physical activity programme assessed in the clinical trial. Many trials do not appear to assess satisfaction with the intervention, and of those that do, reports of these trials tend to describe the measures used poorly. A literature search for measures of satisfaction with the intervention, specifically for physical activity and more generally for use in clinical trials, found one questionnaire which has been properly developed and assessed. However, this questionnaire only assesses satisfaction with exercise classes. Otherwise, measures appear to be developed specifically for each trial, for example, a study trialling an exercise programme in lymphoma survivors used a single question asking participants how they felt about participating in the trial, with participants offered a choice of five answers ('rewarding'; 'waste of my time'; 'useful for research helping others'; 'useful for me personally' and 'something I would recommend to other lymphoma survivors').

Summary and recommendations

Based on a review of the literature and the opinions of experts and lay participants participating in previous stages of this study, we recommend that:

- 'Objective level of physical activity' and 'Health-related quality of life' are included in the physical activity core outcome set.
- Experts and lay participants take a further vote to decide whether to include 'Satisfaction with the intervention' in the physical activity core outcome set.
- There is uncertainty around which is the best device to measure objective level of physical activity. As a minimum, we recommend that an accelerometer is used to measure objective level of physical activity.
- We recommend the EQ-5D is included in the core outcome set as the selected measure to assess 'Health-related quality of life'. It is short, reliable, valid, responsive, can be used for health economic analysis, and is the National Institute for Health and Care Excellence's measure of choice.
- There is currently no appropriate measure to assess satisfaction with physical activity interventions. Therefore, we are unable to recommend a measure for

inclusion in the physical activity core outcome set should this outcome domain be included.

The development of a core outcome set for randomised controlled trials of individual-level physical activity interventions

Background

This project aims to produce a core outcome set for public health trials to promote physical activity in the general adult population, using the standard methods of Core Outcome Sets, namely combining reviews of available evidence and consensus process. Core Outcome Sets have been widely developed and are increasingly influential in clinical research. Their use is intended to produce authoritative agreement on a set of outcome measures to be used in all trials. The result of such standardisation is to make meta-analysis of trials easier and to facilitate the interpretation of available evidence to inform public policy.

The method is designed to reach agreement about two different levels in outcome measurement: (i) broad domains or areas that need to be assessed in trials, and (ii) specific measures that can be recommended for each identified domain or area. In stage one, our consensus process identified two distinct domains that should be assessed in population-level trials to promote physical activity: 'Objective level of physical activity', and 'Health-related quality of life'. A further domain, 'Satisfaction with the intervention', was close to consensus and will therefore be subject to further consideration as part of the remainder of this work.

The second stage of this project focuses on reaching agreement on specific measures for these three domains. This will comprise of a literature review of the selected domains (described further below) followed by a second Delphi survey. Next we discuss the results of stage one, the first Delphi survey, before summarising the literature about the measurement of each of the three identified core domains in turn.

Stage one: Determining the outcome domains

To determine which outcome domains should be included in the core outcome set, we carried out a Delphi survey. In round one, we asked participants to rate the importance of 13 outcome domains commonly measured in clinical trials of physical activity on a scale of 1 to 9, where 1-3 signifies 'limited importance', 4-6 is 'important but not critical' and 7-9 is 'critical importance'. Twenty people took part, of whom 16 were professionals and four were lay participants. At least 70% of participants rated 'Objective level of physical activity' and 'Health-related quality of life' as domains of critical importance (see Table 1) and therefore, they were included in the core outcome set.

In round two, we presented participants with the 11 outcome domains for which no consensus was reached, together with a summary of results from round one, and asked them to re-rate the outcome domains in light of these results. Twenty-one people took part (17 professionals and 4 lay participants). None of the remaining 11 outcome domains gained consensus of at least 70% of participants rating the domain of critical importance (see Table 1). However, 'Satisfaction with the intervention' was close to reaching a consensus with 66.7% rating it as critically important. Therefore, participants will be asked to again consider whether this outcome domain should be included in the core outcome set as part of the second Delphi survey.

Table 1. Results of the first Delphi survey, rounds 1 and 2 - determining which domains to include in the physical activity core outcome set

Domain	Rated 1-3 (limited importance) n (%)	Rated 4-6 (important but not critical) n (%)	Rated 7-9 (critical importance) n (%)	Outcome
Round 1 (n=20[a])				
Level of physical activity (objective)	1 (5.00)	3 (15.00)	16 (80.00)	Consensus – include in COS
Health-related quality of life	0 (0.00%)	6 (30.00)	14 (70.00)	Consensus – include in COS
Other health behaviours	1 (5.00)	5 (26.00)	13 (68.00)	No consensus
Satisfaction with intervention	1 (5.00)	6 (32.00)	12 (63.00)	No consensus
Biophysical health	1 (5.00)	7 (35.00)	12 (60.00)	No consensus
Level of physical activity (self-report)	2 (10.00)	6 (30.00)	12 (60.00)	No consensus
Cost-effectiveness	1 (5.00)	8 (40.00)	11 (55.00)	No consensus
Adverse events	4 (20.00)	5 (25.00)	11 (55.00)	No consensus
Self-efficacy	2 (11.00)	7 (37.00)	10 (53.00)	No consensus
Sedentary behaviour	3 (16.00)	7 (37.00)	9 (47.00)	No consensus
Motivation	1 (5.00)	10 (53.00)	8 (42.00)	No consensus
Physical fitness	2 (11.00)	9 (47.00)	8 (42.00)	No consensus
Physical function	0 (0.00)	12 (63.00)	7 (37.00)	No consensus
Round 2 (n=21)				
Satisfaction with intervention	1 (4.76)	6 (28.57)	14 (66.67)	Borderline no consensus
Physical function	0 (0.00)	10 (47.62)	11 (52.38)	No consensus
Adverse events	1 (4.76)	9 (42.86)	11 (52.38)	No consensus
Sedentary behaviour	4 (19.05)	6 (28.57)	11 (52.38)	No consensus
Cost-effectiveness	1 (4.76)	10 (47.62)	10 (47.62)	No consensus
Biophysical health	2 (9.52)	9 (42.86)	10 (47.62)	No consensus
Level of physical activity (self-report)	4 (19.05)	7 (33.33)	10 (47.62)	No consensus
Other health behaviours	4 (19.05)	7 (33.33)	10 (47.62)	No consensus
Self-efficacy	5 (23.81)	7 (33.33)	9 (42.86)	No consensus
Physical fitness	2 (9.52)	11 (52.38)	8 (38.10)	No consensus
Motivation	3 (14.29)	11 (52.38)	7 (33.33)	No consensus

[a]Note: one individual did not rate all of the outcome domains in the first round due to technical issues

Stage two: Determining measures for the agreed outcome domains

Following the consensus process to determine outcome domains for inclusion in the core outcome set, literature searches were conducted to explore measurement approaches for the three domains that reached or were near to consensus. Each of these domains ('Objective level of physical activity', 'Health-related quality of life', and 'Satisfaction with the intervention') will be discussed next.

Domain 1: Objective level of physical activity

Physical activity is defined as "any bodily movements produced by skeletal muscles that result in energy expenditure" (Caspersen et al., 1985). Physical activity behaviour can be quantified in four ways: intensity; duration; frequency; and type of activity (Strath et al., 2013). Furthermore, the result of physical activity, i.e. energy expenditure, can also be quantified. Physical activity can be measured both subjectively (i.e. through the use of self-report measures) and objectively (i.e. measured by external methods or devices). Here, we discuss the objective measurement of physical activity.

A scoping review was undertaken to identify reviews of the measurement properties of objective measures of physical activity. As measurement technologies are continually improving, the review was limited to the past 10 years (i.e. from 2010 onwards). In addition, primary studies of relevance were also reviewed.

The reviews identified a variety of different measurement approaches and devices for the quantification of physical activity. The accepted gold standard measure of energy expenditure is doubly-labelled water (DLW). However, this method is generally not used in clinical trials as it is expensive, time intensive, and burdensome for the participant (Sylvia et al., 2014; Dowd et al., 2018). Other methods, such as calorimetry, can be expensive and are not practical in free-living environments (Aparicio-Ugarriza et al., 2015; Hills et al., 2014). As such, it would not be feasible to include these measures in a core outcome set. There has been a rapid expansion of types of devices to provide objective measurement, and increasing sophistication in measurement approaches in the use of such devices (Jake-Schoffman et al., 2019). The advantages and disadvantages of a variety of these devices are briefly considered next:

Pedometers
Pedometers measure steps taken, but are unable to measure type of activity, intensity, frequency and duration of physical activity, or allow the calculation of energy expenditure (Aparicio-Ugarriza et al., 2015). They are also unable to measure non-ambulatory activities, such as cycling (Ainsworth et al., 2015). Pedometers are relatively low cost and generally have good accuracy and test-retest reliability (Hills et al., 2014; Ainsworth et al., 2015). However, accuracy can be compromised at slower walking speeds and in those with gait impairments, while also being affected by the placement of the device (Ainsworth et al., 2015; Hills et al., 2014).

Accelerometers
Accelerometers measure accelerations of the body on one or more planes, which can be converted to assess intensity, frequency, and duration of activity, but not type of activity (Hills et al., 2014). The data can also be used to estimate physical activity energy expenditure. Accelerometers tend to perform less well when measuring non-ambulatory activities, low

intensity activities, and sedentary behaviour (Ainsworth et al., 2015). The reliability and validity of accelerometers can be affected by positioning, with hip- and wrist- worn positions considered the most accurate (Ainsworth et al., 2015). Furthermore, measurement properties can vary by model, with newer models having better validity (Sylvia et al., 2014).

Heart rate monitors
Heart rate monitors measure heart rate over time and are able to estimate energy expenditure. They allow the assessment of frequency, intensity and duration of physical activity and are good at capturing data on non-ambulatory activities such as swimming and cycling (Sylvia et al., 2014; Hills et al., 2014). They are a useful tool to use alongside other devices, such as accelerometers, as they are able to capture data that may otherwise be missed, such as carrying a load while walking (Hills et al., 2014). Heart rate monitors are not as accurate at estimating energy expenditure at lower levels of intensity (Ainsworth et al., 2015).

Multi-sensor systems
Multi-sensor systems combine sensors or devices to provide better estimates of physical activity and energy expenditure (Ainsworth et al., 2015; Hills et al., 2014). A multi-sensor system may monitor aspects such as heart rate, accelerometry, respiration, skin temperature, global positioning and so on (Ainsworth et al., 2015). As technology develops, multi-sensor systems will become more advanced and complex.

Measurement issues

Despite increasing sophistication in measurement approaches, there is growing recognition of the nature and scale of measurement issues to be addressed in objective measures of physical activity. Migueles and colleagues (2017) carried out a systematic review of measurement and data collection and processing practices associated with the use of accelerometers. They identified major variations in the following: device placement; sampling frequency; wear-time; what constitutes a valid day and a valid week; cut-points for sedentary time and physical activity intensity classification; and algorithms to estimate physical activity-related energy expenditure. Lacking any gold standard against which to assess observed variations in the use of accelerometers, all the authors could do is to recommend that future researchers are more thoughtful and explicit in their choice of options for the many parameters examined in the review. Jake-Schoffman and colleagues (2019) carried out a similar analysis from a systematic review, but included pedometers and heart-rate monitors as well as accelerometers. Again they found considerable variation in researchers' practices, and pleaded for standardisation, unable to produce evidence for better practice.

This variation in terms of data collection and processing practices has an impact on the interpretation of the data collected (Migueles et al., 2017). For example, Ayabe et al. (2014) found that the treatment of interruptions (i.e. breaks during exercise) affects the estimation of moderate to vigorous intensity physical activity; in a second study (Ayabe et al., 2013) they found that the classification of data into different physical activity groups (i.e. light physical activity, moderate to vigorous intensity physical activity etc.) by epoch length (i.e. the interval at which accelerometer data is recorded) affects the estimation of physical activity; and finally, a study by Robson and Janssen (2015) demonstrate the importance of analysing non-bouted activity (i.e. active periods of less than 10 minutes). The comparability of data

between studies is further complicated by poor reporting of data collection and processing practices (Migueles et al., 2017; Montoye et al., 2018). Montaye and colleagues (2018) have developed a template to encourage the standardisation of the reporting of accelerometer methods.

While many studies analyse data to produce metrics of physical activity, e.g. minutes of moderate to vigorous intensity physical activity per day, an alternative approach is the analysis of physical activity patterns (Shiroma et al., 2018). As analytical approaches to the analysis of accelerometer data rapidly progress, Migueles et al. (2017) recommend the collection of raw accelerometer data in order to maximise the potential for any future data analysis.

As well as a lack of standardisation in the use of devices, no single measurement device can be recommended for all studies (Freedson et al., 2012), but there is a general consensus that the measure selected should be specific to the behaviour of interest and the type of data intended to be collected (Dowd et al., 2018; Sylvia et al., 2014). Strath and colleagues (2013) have produced guidance for selecting optimal methods of assessing physical activity. Such guidance highlights that, when choosing between approaches, it is important to consider practical issues of feasibility and user burden, and financial costs alongside standard issues of reliability and other measurement properties of objective measures. However the focus of this guidance is as much on clinical applications to promote physical activity as on use in population-focused behavioural change trials and evaluative research, potentially limiting its relevance.

Given the proliferation of devices and measurement approaches to objectively assess physical activity, comparative evidence of performance is essential to inform recommendations. Van Remoortel and colleagues (2012) report a systematic review of the validity of activity monitors. They compared the performance of devices, and groups of device, against criteria such as doubly labelled water. Their main grouping of devices was: uniaxial accelerometers, triaxial accelerometers and multisensory devices. Overall evidence of validation was stronger for triaxial accelerometers and multisensory devices compared to uniaxial accelerometers, but they especially stress the high levels of heterogeneity of types of validation study and heterogeneity of results of individual devices within classes or types of device.

Other reviews also found that multi-sensor devices performed better than single devices. A recent systematic review and meta-analysis of wrist- or arm-worn devices was carried out to assess how well they estimate physical activity-related energy expenditure (O'Driscoll et al., 2020). They compared the following grouping of devices: (1) accelerometry alone; (2) heart rate alone; (3) accelerometry and heart rate; (4) accelerometry and heat sensing or galvanic skin response; and (5) accelerometry, heart rate sensors and heat sensing or galvanic skin response sensors. As with other reviews they found considerable heterogeneity of results by specific device and significant variations in performance by type of physical activity being assessed (walking, cycling etc.). Nevertheless there was a clear trend for devices based on accelerometer plus heart rate or accelerometer plus heat sensing to perform significantly better in the assessment of energy expenditure compared to other groupings, for most activities.

With the rapidly expanding range of technologies and associated volume of evidence, reviews of reviews are a valuable adjunct. Dowd and colleagues (2018) have recently carried out such

a review, encompassing 63 reviews in their analyses of technologies to measure physical activity. They present important evidence of the virtual absence of studies that assess responsiveness of instruments, i.e. whether instruments are able to detect change over time in physical activity. Overall however their review of reviews is disappointing in not identifying any clear and consistent pattern of superior performance of any type of objective measure in assessing physical activity.

Systematic reviews, (such as Foster et al., 2013; Stockwell et al., 2019; Silfee et al., 2018) highlight a second and equally important issue to that of the proliferation of devices, namely the variety of constructs (i.e. things being measured) reported in clinical trials of physical activity interventions. Silfee and colleagues (2018) identified 103 studies that used objective measures of physical activity in behavioural trials between 2006 and 2016, and found that by far the commonest thing measured was number of steps, followed by minutes of different levels of activity. Least commonly measured were energy expenditure and accelerometer counts. These reviews show there is fundamental non-comparability of constructs measured between trials, rendering systematic reviews of comparative evidence problematic: to simplify, numbers of steps cannot easily be translated into minutes of particular levels of intense activity and vice-versa. Furthermore, it should be noted that outcomes such as minutes of walking per day and mean hours jogging per week are specific to the behaviour of interest, whereas, outcomes such as energy expenditure are generic and therefore more appropriate for inclusion in a core outcome set.

As already mentioned, there are a number of constructs which devices can be used to estimate. One such construct is physical activity-related energy expenditure (PAEE), which is the component of total energy expenditure that is due to behaviours such as walking and climbing stairs. Total energy expenditure includes other such components such as energy spent from sleeping or being sedentary. With appropriate conversion algorithms, combined measures such as accelerometers and sensors can be used to estimate PAEE. PAEE estimated from wearable devices is a strong predictor of mortality (Strain et al., 2020). Migueles and colleagues (2017) advise caution when interpreting PAEE estimates, as energy expenditure produced from the same movements can vary depending on individuals' characteristics. It is recognised that there remain unresolved issues about details of the most reliable algorithms to estimate PAEE from device data, but there seems to be no evidence that the algorithms have large effects that might produce systematic bias in trials if various algorithms were used.

Summary

There is a clear and consistent trend towards increasing use of objective measures in trials to evaluate interventions to promote physical activity (Silfee et al., 2018). However, there is great heterogeneity in terms of both what is measured as well as by what technology. As such, many reviews conclude that there is a need for consensus around a consistent approach to collecting and reporting data to allow comparisons across instruments (Dowd et al., 2018). Despite increasing pleas to standardise the measurement of physical activity in the light of this proliferation of methods and measures, no standardisation has emerged.

<u>Domain 2: Health-related quality of life</u>

Health-related quality of life (QOL) is a multi-dimensional concept with typical dimensions including physical functioning, psychological well-being, social functioning, symptoms and role activities. QOL is subjective to each individual and therefore needs to be measured by

self-report. This usually takes the form of patient-reported outcome measures (PROMs) which are short questionnaires that ask people to report their views on a range of quality of life issues. Broadly, PROMs fall into two categories: generic or disease-specific. Generic measures are applicable to everyone, whereas disease specific measures are only relevant to a person with the specified disease or condition. Generic measures therefore have wider applicability as they are relevant in the healthy population, and in those with a health problem including one or multiple morbidities. Disease-specific measures are, however, more sensitive, and are likely to assess more relevant issues for a particular disease or condition. The Core Outcome Set (COS) for physical activity is intended to be relevant for all adults; hence, quality of life in this context could only be assessed by a generic measure to allow for the widest possible applicability. An additional benefit of using a generic measure is that the most commonly used measures are well validated and tested in a wide range of populations. They would therefore be suitable for use in the context of a COS in physical activity.

A pragmatic scoping review was undertaken to identify the most relevant and widely used generic PROMs in physical activity interventions. The initial step was to identify reviews on quality of life in relation to physical activity. Of the reviews identified many were over 10 years old (e.g. Rejeski and Mihalko, 2001; Bize et al., 2007). No review comprehensively assessed quality of life measurement in physical activity trials. Instead, identified reviews focused on describing the relationship between quality of life and physical activity, often in a specified disease or age group. Some examples are drawn on here. The probably most relevant review was by Bize et al. (2007), as it focused on the general population. This review of cross-sectional and cohort studies, RCTs and mixed designs identified the SF-36 and its derivatives as the most used health-related quality of life instruments used in physical activity in the general population (Bize et al., 2007). None of the studies in the Bize et al. (2007) review had used the EQ-5D. The review by Rejeski and Mihalko (2001) focused on the relationship of quality of life and physical activity of older adults. They do list the instruments used in the included studies with the SF-36 and the Sickness Impact Profile being the most commonly listed. Two reviews on physical activity in cancer identified the EORTC QLQ-30 (cancer-specific measure) as the most used instrument, with the SF-36 the most commonly used generic measure (Mishra et al., 2012; Buffart et al., 2017). A meta-analysis of the effect of exercise on physical function, activities of daily living and quality of life in the frail elderly only included trials that had used the SF-36 (Chou et al., 2012).

As these reviews are unlikely to give the full picture of the most appropriate generic PROMs in physical activity trials, additional searching on primary research was conducted. Trials usually only assessed quality of life when the study population were the frail elderly or people with a chronic disease (even when specifically searching for 'healthy' or 'general' population, the results yielded were mostly trials in populations who were older or who had chronic disease). Chronic disease is more common in the older population (e.g. 58% of those aged over 60 have a chronic condition), but 14% of those under 40 years of age have at least one chronic condition (Department of Health and Social Care, 2012). Due to the smaller number of physical activity interventions assessing quality of life in the younger and/or healthy population being lower than expected; the search was expanded to including other types of study, such as cross-sectional or cohort-type designs to gain a view of the most widely used generic PROM. Searches were also conducted to identify physical activity trials that have used the best known generic measures. These searches identified that two of the most widely used generic quality of life measures (i.e. the Short-Form 36 (SF-36) (Ware and Sherbourne, 1992),

and its derivatives such as the SF-12 (Ware, 1995), and the EuroQOL EQ-5D (EuroQol, 1990) also appear to be the most widely used generic measures in physical activity trials. Other generic measures, such as the Sickness Impact Profile (Bergnet et al., 1976), WHO QOL-BREF (The WHOQOL Group, 1998) or Health Utility Index (Horsman et al., 2003), are not considered any further as they are not commonly used in physical activity trials. Hence, the two main contenders for the COS are the SF-36 and EQ-5D and they are in turn briefly described.

The SF-36 (Ware and Sherbourne, 1992), developed in the US, is a 36-item measure intended for use in the general population and a wide range of conditions). The items are scored to give eight domains (bodily pain; general health perceptions; mental health; physical functioning; role limitations due to emotional health problems; role limitations due to physical health problems; social functioning; and vitality). A physical component score and mental component score can also be derived from the items (Ware, Kosinski, et al., 1994). The domain and component scores range from 0-100 with higher scores meaning better quality of life. The SF-36 has been very widely validated in a range of populations (e.g. Ware, Gandek et al. (1994) in mental health; Gandek et al. (2004) in elderly and disabled; Obidoa et al. (2010) in healthy population) and different countries (e.g. Stewart et al. (1988) in the US; Stansfeld et al. (1997) in the UK) and is found to be valid and reliable.

Amongst the derivatives of the SF-36 is the SF-12 which was produced in response to a need of shorter instruments (Ware, 1995). The 12 items are scored into a Physical Component Score and Mental Component score, ranging from 0-100 with higher scores depicting better quality of life. The SF-12 has been shown to be valid in a UK population (Jenkinson and Layte, 1997) and to be an adequate substitute for the SF-36 as scores between the two instruments are virtually identical (Jenkinson et al., 1997). The SF-6D version (Brazier et al., 2002) was developed for health economic analysis, however there are some concerns over inconsistent estimates and prediction values of the poorest health states. The SF-12 is used in physical activity studies but considerably less frequently than the SF-36, whilst the SF-6D has been used rarely in physical activity interventions.

The EQ-5D (EuroQol, 1990), developed in five European Countries, is a 5-item health status measure together with a Visual Analogue Scale (VAS). Each item represents a domain (anxiety/depression; pain; self-care; mobility; and usual activities). A score ranging from 0-1 is calculated from the items, with a higher score meaning better quality of life (NB. It is possible to score below 0, indicating a health state worth than death). The VAS ranges from 0-100, with higher scores also indicating better quality of life. Similarly to the SF-36, the EQ-5D has been very widely validated in different population groups (e.g. Brazier et al. (1993) in the general population, Hurst et al. (1994) in rheumatoid arthritis) and in different countries (e.g. Brazier et al. (1993) in the UK, Nord et al. (1991) in Norway), and a recent systematic review concludes that it has excellent psychometric properties (Feng et al., 2020). The EQ-5D can be used in health economic analysis (e.g. Deidda et al. (2018) cost-effectiveness of exercise referral scheme in older adults) and value sets have been published for many countries including the UK (Dolan, 1997; Devlin et al., 2018). It is also the measure of choice for the National Institute for Health and Care Excellence (NICE) (National Institute of Health and Social Care, 2019).

Overall, the conclusion is that the SF-36 is more widely used in physical activity clinical trials. However, it is a longer instrument than the EQ-5D and therefore more burdensome for trial

participants. The EQ-5D is shorter, is valid for health economic analysis, and is the measure of choice for NICE. Therefore, the EQ-5D is the recommended measure.

Domain 3: Satisfaction with the intervention

This domain intends to capture the extent to which study participants experience and perceive the physical activity intervention as positive. It is thought that understanding the experience of people who have participated in a physical activity programme is key to understanding their motivation, engagement, and compliance with the programme (Slade and Keating, 2010). Following the second round of the consensus process, 66.7% of stakeholders rated the 'Satisfaction with intervention' domain as critically important (i.e. 7 – 9), with 4.8% rating it as of limited importance (i.e. 1 – 3). As the domain was close to reaching a consensus, further details on satisfaction with the intervention are given here. A further round of consultation will seek to identify whether this domain should be included, and if so, to gain views on a measure to use.

It is challenging to identify studies that assess satisfaction with the intervention. Searches for this scoping review yielded studies that focus on measuring self-reported physical activity (e.g. Falk et al., 2016; Dowd et al., 2018) or other types of satisfaction (predominantly 'life satisfaction' but also issues such as 'job satisfaction'). Searches on 'experiences with the intervention' mostly identifies measures of physical activity, which are part of a different domain. Adherence to the intervention could be considered a proxy measure for satisfaction. However aside from being conceptually different and this research not having sought consensus on adherence, there is no agreement on how adherence should be measured (Hawley-Hague et al., 2016; McLean et al., 2017; Levy et al., 2019). Reviews focus on specific sub-samples of the population e.g. older population (Hawley-Hague et al., 2016), musculoskeletal settings (McLean et al., 2017) and stroke (Levy et al., 2019) and can also focus on a specific type of exercise (e.g. Hawley-Hague et al. (2016) reviewed measures used for assessing adherence to exercise classes). Broadening the search to trials that do not focus on physical activity, to identify a generic method of measuring satisfaction with an intervention, showed that trials that assess satisfaction with the intervention predominantly use their own measure. Frequently, these measures are not well described in the publications (for both physical activity trials and other types of trials).

Despite these challenges, the scoping review revealed one review (Slade and Keating, 2010) and one physical activity satisfaction measure (Cunningham, 2007). Additionally, the search found that while some trials have assessed satisfaction with the intervention (e.g. Hawkes et al., 2014; Pebole and Hall, 2019) many trials do not (e.g. Schulz et al., 2014; Barrett et al., 2018; Wou et al., 2018). The review (Slade and Keating, 2010) focused on measuring participant experience and satisfaction of exercise programmes for low back pain and identified 10 potentially relevant instruments. However, these were physiotherapy type exercise interventions, rather than public health ones, and the measures tended to focus on the quality of care provided rather than satisfaction with the intervention. Some satisfaction with the intervention measures with higher relevance were identified. However, they tended to be specific to the study they were used in, usually in a specific condition or population. The satisfaction measure, although developed and tested appropriately, was targeted at satisfaction with physical activity classes (Cunningham, 2007) and was developed in a student population. No evidence was found that this measure was validated for use in other

populations; and even if it had, it would be too limited for our core outcome set as it does not solely focus on exercise classes.

Three examples of trials are given to illustrate the range of measures used. The multi-behaviour intervention (including physical activity) in colorectal cancer by Hawkes and colleagues Hawkes et al. (2014) used three satisfaction questions, the first on whether the intervention met the participant's expectations; the second whether the intervention helped the participant achieve their goals; and the third on overall satisfaction with the intervention. All questions were rated on a 4 point scale.

Courneya et al. (2013) conducted an exercise trial in lymphoma survivors and measured satisfaction with one question on how participants felt about participating in the trial with 5 response options ('rewarding'; 'waste of my time'; 'useful for research helping others'; 'useful for me personally' and 'something I would recommend to other lymphoma survivors'). Further questions focused on how they felt about their trial group assignment, and the burden of the tests associated with the trial. There were also some further questions specific to each trial arm (e.g. on perceived barriers and support). The authors highlight that they developed their own satisfaction measure as none are available and acknowledge the lack of a validated measure as a limitation for the study.

Another example is an exercise intervention in older veterans with PTSD (Pebole and Hall, 2019) in which participants were asked to complete a program evaluation upon trial completion. This included targeted and open-ended questions about their experience with the program, perceptions of the exercise environment (physical and social), and preferences for the content and delivery of similar, future exercise programs. For example, one item asked for level of agreement with the statement "I felt comfortable in the exercise setting".

As indicated above, and demonstrated by these examples, measures of satisfaction with a physical activity exercise intervention are used in some trials but these tend to be mostly specific to each trial. Aside from one example of a measure developed to assess satisfaction with exercise classes (Cunningham, 2007), no information is available on how these measures have been developed and validated. It follows that none of these measures have been used widely. In the absence of any psychometric information on any of these measures, no specific measure can be recommended. If there is consensus that this domain should be included in a core outcome set for physical activity, a new measure will have to be developed for this purpose.

Summary and recommendations

The purpose of this study is to develop a core outcome set for physical activity clinical trials. Stage one of the study aimed to identify which outcome domains should be measured, with a consensus approach identifying 'Objective level of physical activity' and 'Health-related quality of life' as core domains for inclusion in the core outcome set. There was some uncertainty around the inclusion of a third domain, 'Satisfaction with the intervention', and therefore consensus will be sought in the next stage of the study. Stage two of the study aims to reach agreement about how to measure the included outcome domains. A literature review identifying and evaluating the measurement tools available was carried out for each outcome domain. A second Delphi survey will now be undertaken to reach consensus about the most appropriate measurement tools to include in the core outcome set, for each of the

agreed domains. Recommendations based on the first Delphi survey and literature review are set out in Box 1 below.

Box 1. Recommendations for the physical activity core outcome set

- ‘Objective level of physical activity’ and ‘Health-related quality of life’ should be included as core outcome domains in the physical activity core outcome set.
- Consensus has yet to be reached about whether to include ‘Satisfaction with the intervention’ as an outcome domain in the physical activity core outcome set. We will conduct a further round of voting to determine whether or not to include it.
- There is uncertainty around which is the best device to measure ‘Objective level of physical activity’. As a minimum, we recommend that this domain is measured using an accelerometer.
- We recommend the EQ-5D is included in the core outcome set as the selected measure to assess health-related quality of life. It is short, reliable, valid, responsive, can be used for health economic analysis, and is the National Institute for Health and Care Excellence’s measure of choice.
- There are currently no well-developed and validated measures that can be used to assess satisfaction with physical activity interventions. Therefore, we are unable to recommend a measure for inclusion in the physical activity core outcome set should this outcome domain be included.

References

Ainsworth, B., Cahalin, L., Buman, M. & Ross, R. (2015). The current state of physical activity assessment tools. *Progress in Cardiovascular Diseases,* 57**,** 387-95.

Aparicio-Ugarriza, R., Mielgo-Ayuso, J., Benito, P. J., Pedrero-Chamizo, R., Ara, I., Gonzalez-Gross, M. et al. (2015). Physical activity assessment in the general population; instrumental methods and new technologies. *Nutricion Hospitalaria,* 31 Suppl 3**,** 219-26.

Ayabe, M., Kumahara, H., Morimura, K. & Tanaka, H. (2013). Epoch length and the physical activity bout analysis: an accelerometry research issue. *BMC Research Notes,* 6**,** 20.

Ayabe, M., Kumahara, H., Morimura, K. & Tanaka, H. (2014). Interruption in physical activity bout analysis: an accelerometry research issue. *BMC Research Notes,* 7**,** 284.

Barrett, S., Begg, S., O'Halloran, P. & Kingsley, M. (2018). Integrated motivational interviewing and cognitive behaviour therapy can increase physical activity and improve health of adult ambulatory care patients in a regional hospital: the Healthy4U randomised controlled trial. *BMC Public Health,* 18**,** 1166.

Bergnet, M., Bobbitt, R. A., Pollard, W. E., Martin, D. P. & Gilson, B. S. (1976). The sickness impact profile: validation of a health status measure. *Medical Care,* 14**,** 57-67.

Bize, R., Johnson, J. A. & Plotnikoff, R. C. (2007). Physical activity level and health-related quality of life in the general adult population: a systematic review. *Preventive Medicine,* 45**,** 401-15.

Brazier, J., Jones, N. & Kind, P. (1993). Testing the validity of the Euroqol and comparing it with the SF-36 health survey questionnaire. *Quality of Life Research,* 2**,** 169-80.

Brazier, J., Roberts, J. & Deverill, M. (2002). The estimation of a preference-based measure of health from the SF-36. *Journal of Health Economics,* 21**,** 271-92.

Buffart, L. M., Kalter, J., Sweegers, M. G., Courneya, K. S., Newton, R. U., Aaronson, N. K. et al. (2017). Effects and moderators of exercise on quality of life and physical function in patients with cancer: An individual patient data meta-analysis of 34 RCTs. *Cancer Treatment Reviews,* 52**,** 91-104.

Caspersen, C. J., Powell, K. E. & Christenson, G. M. (1985). Physical activity, exercise, and physical fitness: definitions and distinctions for health-related research. *Public Health Reports,* 100**,** 126-31.

Chou, C. H., Hwang, C. L. & Wu, Y. T. (2012). Effect of exercise on physical function, daily living activities, and quality of life in the frail older adults: a meta-analysis. *Archives of Physical Medicine and Rehabilitation,* 93**,** 237-44.

Courneya, K. S., Forbes, C. C., Trinh, L., Sellar, C. M., Friedenreich, C. M. & Reiman, T. (2013). Patient satisfaction with participation in a randomized exercise trial: effects of randomization and a usual care posttrial exercise program. *Clinical Trials,* 10**,** 959-66.

Cunningham, G. (2007). Development of the Physical Activity Class Satisfaction Questionnaire (PACSQ). *Measurement in Physical Education and Exercise Science,* 11**,** 161-176.

Deidda, M., Coll-Planas, L., Gine-Garriga, M., Guerra-Balic, M., Roque, I. F. M., Tully, M. A. et al. (2018). Cost-effectiveness of exercise referral schemes enhanced by self-management strategies to battle sedentary behaviour in older adults: protocol for an economic evaluation alongside the SITLESS three-armed pragmatic randomised controlled trial. *BMJ Open,* 8**,** e022266.

Department of Health and Social Care. (2012). https://www.gov.uk/government/publications/long-term-conditions-compendium-

of-information-third-edition. [Online]. Available at: http://www.dh.gov.uk/publications (accessed: 22 March 2021).

Devlin, N. J., Shah, K. K., Feng, Y., Mulhern, B. & Van Hout, B. (2018). Valuing health-related quality of life: An EQ-5D-5L value set for England. *Health Economics,* 27**,** 7-22.

Dolan, P. (1997). Modeling valuations for EuroQol health states. *Medical Care,* 35**,** 1095-108.

Dowd, K. P., Szeklicki, R., Minetto, M. A., Murphy, M. H., Polito, A., Ghigo, E., et al. (2018). A systematic literature review of reviews on techniques for physical activity measurement in adults: a DEDIPAC study. *International Journal of Behavioral Nutrition and Physical Activity,* 15**,** 15.

EuroQol. (1990). EuroQol - a new facility for the measurement of health-related quality of life. *Health Policy,* 16**,** 199-208.

Falk, R. S., McDonald, S. M., Beets, M. W., Brazendale, K. & Lui-Ambrose, T. (2016). Measurement of physical activity in older adult interventions: a systematic review. *British Journal of Sports Medicine,* 50**,** 464-470.

Feng, Y. S., Kohlmann, T., Janssen, M. F. & Buchholz, I. (2020). Psychometric properties of the EQ-5D-5L: a systematic review of the literature. *Quality of Life Research*. https://doi.org/10.1007/s11136-020-02688-y.

Foster, C., Richards, J., Thorogood, M. & Hillsdon, M. (2013). Remote and web 2.0 interventions for promoting physical activity. *Cochrane Database of Systematic Reviews,* 9**,** CD010395.

Freedson, P., Bowles, H. R., Troiano, R. & Haskell, W. (2012). Assessment of physical activity using wearable monitors: recommendations for monitor calibration and use in the field. *Medicine & Science in Sports & Exercise,* 44**,** S1-4.

Gandek, B., Sinclair, S. J., Kosinski, M. & Ware, J. E. (2004). Psychometric evaluation of the SF-36 health survey in Medicare managed care. *Health Care Financing Review,* 25**,** 5-25.

Hawkes, A. L., Pakenham, K. I., Chambers, S. K., Patrao, T. A. & Courneya, K. S. (2014). Effects of a multiple health behavior change intervention for colorectal cancer survivors on psychosocial outcomes and quality of life: a randomized controlled trial. *Annals of Behavioral Medicine,* 48**,** 359-70.

Hawley-Hague, H., Home, M., Skelton, D. A. & Todd, C. (2016). Review of how we should define (and measure) adherence in studies examining older adults' participation in exercise classes. *BMJ Open*. doi: 10.1136/bmjopen-2016-011560.

Hills, A. P., Mokhtar, N. & Byrne, N. M. (2014). Assessment of physical activity and energy expenditure: an overview of objective measures. *Frontiers in Nutrition,* 1**,** 5.

Horsman, J., Furlong, W., Feeny, D. & Torrance, G. (2003). The Health Utilities Index (HUI): concepts, measurement properties and applications. *Health and Quality of Life Outcomes,* 1**,** 54.

Hurst, N. P., Jobanputra, P., Hunter, M., Lambert, M., Lochhead, A. & Brown, H. (1994). Validity of Euroqol - a generic health status instrument - in patients with rheumatoid arthritis. *British Journal of Rheumatology,* 33**,** 655-62.

Jake-Schoffman, D., Silfee, V. & Sreedhara, M. (2019). Reporting of physical activity device measurement and analysis protocols in lifestyle interventions. *American Journal of Lifestyle Medicine*. doi: https://doi.org/10.1177/1559827619862179

Jenkinson, C. & Layte, R. (1997). Development and testing of the UK SF-12 (short form health survey). *Journal of Health Services Research & Policy,* 2**,** 14-8.

Jenkinson, C., Layte, R., Jenkinson, D., Lawrence, K., Petersen, S., Paice, C. et al. (1997). A shorter form health survey: can the SF-12 replicate results from the SF-36 in longitudinal studies? *Journal of Public Health Medicine,* 19**,** 179-86.

Levy, T., Laver, K., Killington, M., Lannin, N. & Crotty, M. (2019). A systematic review of measures of adherence to physical exercise recommendations in people with stroke. *Clinical Rehabilitation,* 33**,** 535-545.

McLean, S., Holden, M. A., Potia, T., Gee, M., Mallett, R., Bhanbhro, S., et al. (2017). Quality and acceptability of measures of exercise adherence in musculoskeletal settings: a systematic review. *Rheumatology,* 56**,** 426-438.

Migueles, J. H., Cadenas-Sanchez, C., Ekelund, U., Delisle Nystrom, C., Mora-Gonzalez, J., Lof, M. et al. (2017). Accelerometer data collection and processing criteria to assess physical activity and other outcomes: A systematic review and practical considerations. *Sports Medicine,* 47**,** 1821-1845.

Mishra, S. I., Scherer, R. W., Snyder, C., Geigle, P. M., Berlanstein, D. R. & Topaloglu, O. (2012). Exercise interventions on health-related quality of life for people with cancer during active treatment. *Clin Otolaryngol,* 37**,** 390-2.

Montoye, A. H. K., Moore, R. W., Bowles, H. R., Korycinski, R. & Pfeiffer, K. A. (2018). Reporting accelerometer methods in physical activity intervention studies: a systematic review and recommendations for authors. *British Journal of Sports Medicine,* 52**,** 1507-1516.

National Institute of Health and Social Care. (2019). Position statement on use of the EQ-5D-5L value set for England (updated October 2019). Available at: https://www.nice.org.uk/about/what-we-do/our-programmes/nice-guidance/technology-appraisal-guidance/eq-5d-5l (accessed: 22 March 2021).

Nord, E. (1991). EuroQol: health-related quality of life measurement. Valuations of health states by the general public in Norway. *Health Policy,* 18**,** 25-36.

O'Driscoll, R., Turicchi, J. & Beaulieu, J. (2020). How well do activity monitors estimate energy expenditure? A systematic review and meta-analysis of the validity of current technologies. *British Journal of Sports Medicine,* 54**,** 332-340.

Obidoa, C. A., Reisine, S. L. & Cherniak, M. (2010). How does the SF-36 perform in healthy populations? A structured review of longitudinal studies. *Journal of Social, Behavioral, and Health Sciences,* 4**,** 30-48.

Pebole, M. M. & Hall, K. S. (2019). Insights following implementation of an exercise intervention in older veterans with PTSD. *International Journal of Environmental Research and Public Health,* 16.

Rejeski, W. J. & Mihalko, S. L. (2001). Physical activity and quality of life in older adults. *Journals of Gerontology. Series A Biological Sciences and Medical Sciences,* 56 Special Issue 2**,** 23-35.

Robson, J. & Janssen, I. (2015). Intensity of bouted and sporadic physical activity and the metabolic syndrome in adults. *PeerJ,* 3**,** e1437.

Schulz, D. N., Kremers, S. P., Vandelanotte, C., Van Adrichem, M. J., Schneider, F., Candel, M. J. et al. (2014). Effects of a web-based tailored multiple-lifestyle intervention for adults: a two-year randomized controlled trial comparing sequential and simultaneous delivery modes. *Journal of Medical Internet Research,* 16.

Shiroma, E. J., Schrack, J. A. & Harris, T. B. (2018). Accelerating accelerometer research in aging. *Journals of Gerontology, Series A Biological Sciences and Medical Sciences,* 73**,** 619-621.

Silfee, V. J., Haughton, C. F., Jake-Schoffman, D. E., Lopaz-Cepero, A., May, C. N., Sreedhara, M., et al. (2018). Objective measurement of physical activity outcomes in lifestyle interventions among adults: A systematic review. *Preventive Medicine Reports,* 11**,** 74-80.

Slade, S. C. & Keating, J. L. (2010). Measurement of participant experience and satisfaction of exercise programs for low back pain: a structured literature review. *Pain Medicine,* 11**,** 1489-99.

Stansfeld, S. A., Roberts, R. & Foot, S. P. (1997). Assessing the validity of the SF-36 General Health Survey. *Quality of Life Research,* 6**,** 217-24.

Stewart, A. L., Hays, R. D. & Ware, J. E. (1988). The MOS short-form general health survey. Reliability and validity in a patient population. *Medical Care,* 26**,** 724-35.

Stockwell, S., Schofield, P., Fisher, A., Firth, J., Jackson, S. E., Stubbs, et al. (2019). Digital behavior change interventions to promote physical activity and/or reduce sedentary behavior in older adults: A systematic review and meta-analysis. *Experimental Gerontology,* 120**,** 68-87.

Strain, T., Wijndaele, K., Dempsey, P. C., Sharp, S. J., Pearce, M., Jeon, J. et al. (2020). Wearable-device-measured physical activity and future health risk. *Nature Medicine,* 26**,** 1385-1391.

Strath, S. J., Kaminsky, L. A., Ainsworth, B. E., Ekelund, U., Freedson, P. S., Gary, R. A., et al. (2013). Guide to the assessment of physical activity: Clinical and research applications: a scientific statement from the American Heart Association. *Circulation,* 128**,** 2259-79.

Sylvia, L. G., Bernstein, E. E., Hubbard, J. L., Keating, L. & Anderson, E. J. (2014). Practical guide to measuring physical activity. *Journal of the Academy of Nutrition and Dietetics,* 114**,** 199-208.

The WHOQOL Group. (1998). Development of the World Health Organization WHOQOL-BREF quality of life assessment. *Psychological Medicine,* 28**,** 551-8.

Van Remoortel, H., Giavedoni, S., Raste, Y., Burtin, C., Louvaris, Z., Gimeno-Santos, E., et al. (2012). Validity of activity monitors in health and chronic disease: a systematic review. *International Journal of Behavioral Nutrition and Physical Activity,* 9**,** 84.

Ware, J. E., Gandek, B., & and IQOLA Project Group. (1994). The SF-36 health survey: development and use in mental health research and the IQOLA project. *International Journal of Mental Health*, 23, 49-73.

Ware, J., Kosinski, M., & Keller, S. D. (1994). SF-36 Physical and Mental Health Summary Scales: a user's manual. Boston, MA: The Health Institute: New England Medical Centre.

Ware, J. (1995). SF-12: How to score the SF-12 Physical and Mental Health Summary Scales. The Health Institute (Second Edition), New England Medical Center. Boston, MA.

Ware, J. E. & Sherbourne, C. D. (1992). The MOS 36-item short-form health survey (SF-36). Conceptual framework and item selection. *Medical Care,* 30**,** 473-83.

Wou, C., Silarova, B., Griffin, S. & Usher-Smith, J. A. (2018). The associations between the response efficacy and objective and subjective change in physical activity and diet in the Information and Risk Modification trial. *Public Health,* 165**,** 26-33.
